# No species loss, but pronounced species turnover in grasslands in the Northern Alps over 25 years

**DOI:** 10.1111/avsc.12700

**Published:** 2022-12-18

**Authors:** Helena Schwaiger, Bernd Lenzer, Franz Essl

**Affiliations:** ^1^ BioInvasions, Global Change, Macroecology Group, Department of Botany and Biodiversity Research University of Vienna Vienna Austria

**Keywords:** alien species, Alps, Ellenberg Indicator Values, grasslands, land‐use change, plant‐species richness, protected areas, resurvey, species loss

## Abstract

The abandonment of marginally productive habitats and the intensification of land use on productive sites have caused transformative changes in vegetation composition in Central Europe. In this study, after 25 years we resurveyed a total of 145 grassland relevés from the mid‐1990s in a grassland‐dominated valley of the Northern Alps of Upper Austria. We studied changes in richness and composition, and related these to underlying drivers. We found that the average species number in plots increased from 46 in the first survey period to 49 in the second one. Median species richness across sites significantly increased from 1995 to 2020 for *Festuco‐Brometea* (55–61 species) and *Galio‐Urticetea* (24–32 species), but did not show any significant change for the other classes. Further, we recorded substantial species turnover, with winners consisting mostly of species that prefer nutrient‐rich sites, while losers were predominantly species of nutrient‐poor sites. In particular, using Ellenberg Indicator Values for calculating community indices, we found an indication for ongoing eutrophication in vegetation types of nutrient‐poor vegetation classes ( *Festuco‐Brometea* and *Calluno‐Ulicetea* ), and in wet habitats (*Scheuchzerio‐Caricetea fuscae*). Community indices of wet habitats also showed clear signs of becoming more mesic. Thermophilization of community indices was evident across several vegetation classes. Further, alien species that were very rare in the mid‐1990s became more abundant in the resurveyed plots, although the level of invasion is still low. Finally, community values for nutrients of plots that are located in a protected area that has been established in 2014 did not increase significantly, while this was the case in plots outside the protected area, indicating that the management of the protected area has positive effects in halting eutrophication. We conclude that despite overall species richness changing only moderately between both surveys, substantial changes in community composition toward more nitrophilic and thermophilic conditions occurred.

## Graphical abstract

We resurveyed a total of 145 grassland relevés from the mid‐1990s in the Northern Alps of Upper Austria. We found that the average species number in plots increased from 46 in the first survey to 49 in the second one. We recorded substantial species turnover, with winners consisting mostly of species that prefer nutrient‐rich sites.

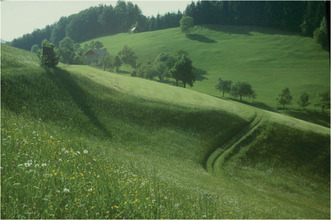

## 1 INTRODUCTION

The increase of anthropogenic pressures such as land‐use changes, pollution, climate change, direct exploitation, and the introduction of invasive alien species causes widespread species declines worldwide, and in Europe in particular (e.g. Mihoub et al., 2017; Diaz et al., 2019; IPBES, 2019). For plants, however, studies on small spatial scales (e.g. using resampled plots) showed divergent results in changes of species richness over time in Europe. While many studies found a decline in plant‐species richness at small scales during the last decades (e.g. Wesche et al., 2012; Hülber et al., 2017; Jansen et al., 2019; Eichenberg et al., 2021), in some cases no net change in species richness was recorded (e.g., Vellend et al., 2013; Diekmann et al., 2014; Ridding et al., 2021), or species richness even increased (e.g., Bühler & Roth, 2011; McCune & Vellend, 2013; Mitchell et al., 2017; Finderup Nielsen et al., 2019).

The decline of plant species is associated with several interacting anthropogenic drivers. In Europe, increasing eutrophication via fertilization and airborne nitrogen deposition is an important driver of plant‐species loss, particularly in nutrient‐poor habitats (e.g. Bobbink et al., 2015). Similarly, other facets of land‐use intensification (e.g. herbicide application, frequent cuts of grasslands, drainage) are known to contribute to plant‐species‐richness losses. The resulting increasing fragmentation of many habitats of high plant‐species richness causes detrimental impacts on species survival due to isolation and Allee‐effects in remaining habitat patches (Jansen et al., 2019). Taken together, many plant species declined in distribution and abundance in recent decades (Kempel et al., 2020; Zehm et al., 2020; Jandt et al., 2022), although in some cases conservation efforts have halted or reversed these declines (Bruelheide et al., 2020).

However, despite somewhat heterogeneous trends in the net change of plant‐species richness at small scales, the turnover of plant species in communities is substantially more pronounced: for instance, there is mounting evidence of changing plant‐community composition towards species with high temperature requirements (= thermophilization, e.g. Gottfried et al., 2012) and high nutrient‐demands (= eutropication, e.g. Hülber et al., 2017; Peppler‐Lisbach et al., 2020). Further, gains and losses often differ between native and alien species (Cardinale, 2018), and accordingly, the spread of new alien species can putatively (partly) compensate for the loss of native species. As a result, plant‐community compositions often become more similar (Bühler & Roth, 2011; Finderup Nielsen et al., 2019).

European grasslands have been created over more than 1000 years by extensive land use (grazing, mowing) in a wide range of different sites (Tälle et al., 2016); consequently, a wide range of different grassland habitats has developed (Habel et al., 2013; Batáry et al., 2015; Janssen et al., 2016). For grasslands in Europe, the intensification of land use is one of the major drivers of plant biodiversity loss with cascading effects on other taxonomic groups such as arthropods (Gossner et al., 2016). Further, while the level of invasion of aliens into grasslands is still relatively low in Europe, it is increasing (Axmanová et al., 2021). In addition, the abandonment of marginal, yet often extensively used and thus species‐rich grasslands, also has substantial negative impacts on grassland biodiversity (Diekmann et al., 2014; Halada et al., 2017). Thus, maintaining low‐intensity farming is crucial for the conservation of European grassland biodiversity (Sutcliffe et al., 2015). Although numerous grassland habitats are protected by the European Habitats Directive (European Commission, 2007), nutrient‐poor grasslands severely decreased in the last decades (Hülber et al., 2017).

Here, we assessed the changes in the grassland vegetation in a pre‐alpine landscape in Austria — the Jaidhaus basin — over 25 years by resurveying 145 plots in 2020 that had been first sampled in 1995. In particular, we address the following researcfh questions: (1) how did plant‐species richness and community composition (e.g. in terms of Ellenberg Indicator Values) change over the last 25 years? (2) Did the trajectories in vegetation composition and species turnover differ among different grassland habitats? (3) What are the impacts of land use, land‐use changes and of the recent establishment of a protected area on changes in species richness and community composition? (4) Did alien plant species spread during the last 25 years?

## 2 METHODS

### 2.1 Study area

**FIGURE 1:**
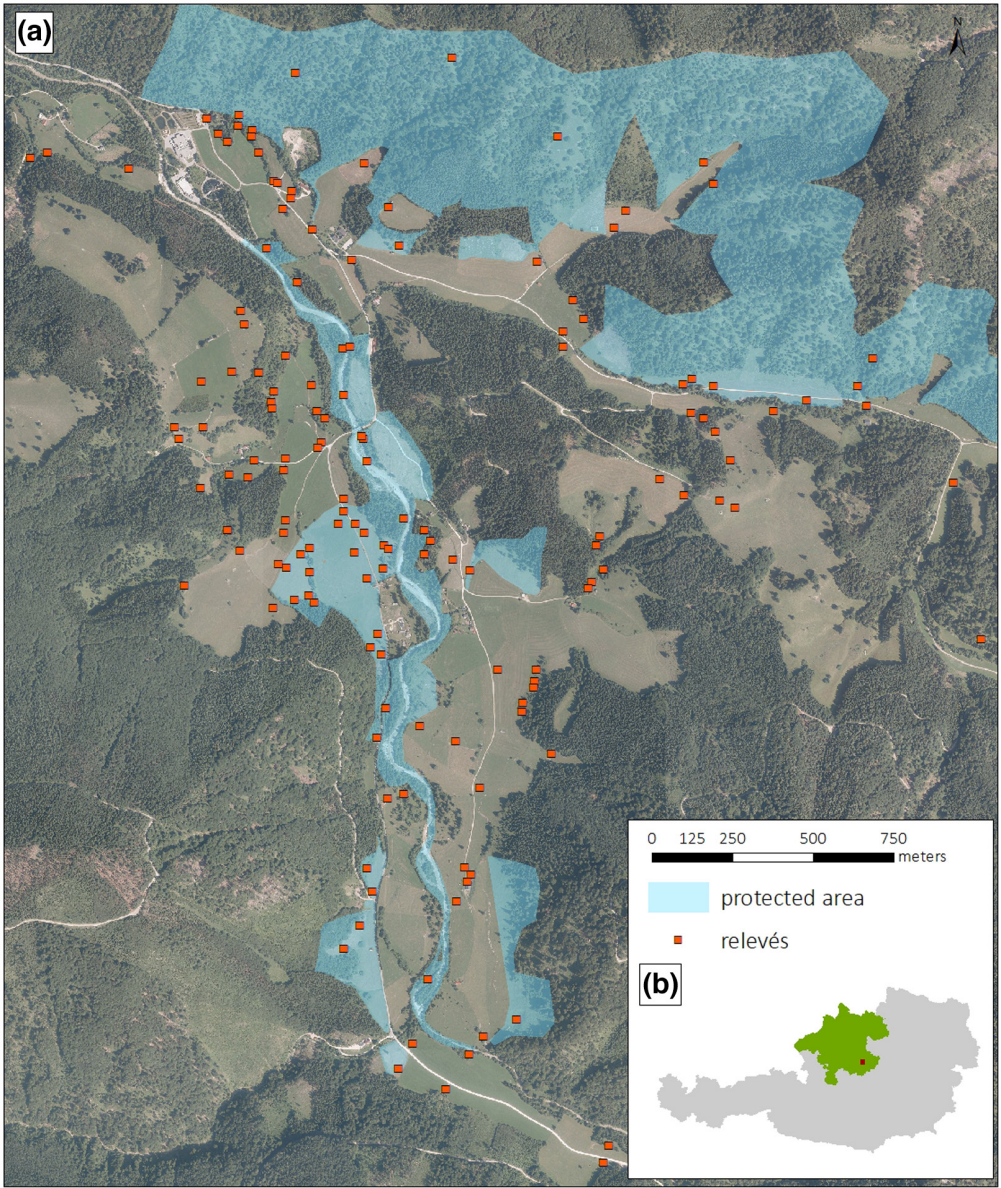
(a) Overview of the study area Jaidhaus basin including the nature reserve (blue shading) and the sampling plots. (b) Location of the study area in Austria (grey) and Upper Austria (green). Source aerial images: ESRI Basemap; source protected area: open government data Upper Austria

The study area — the Jaidhaus basin (c. 5 km^2^) — is located at the Krumme Steyling river in the pre‐Alps near the village of Molln in southeastern Upper Austria. The study area is dominated by a range of different submontane grassland types (510–720 m a.s.l.) and surrounded by mid‐altitude mountains (Figure 1). A substantial part of former grasslands has been abandoned during the last decades, and forest cover has increased accordingly (Priller, 2014; Figure S1). The study area belongs to the Northern Calcareous Alps, and bedrock mostly consists of limestone, dolomite and calcareous gravel at the valley bottom. The Jaidhaus basin is characterized by a temperate suboceanic montane climate. The nearest weather station (Windischgarsten, 600 m a.s.l.) reports annual mean precipitation of 1443 mm (1991–2010). The annual mean temperature has increased considerably during the last decade from 7.4°C (1991–2010) to 8.9°C (2011–2020), that is, an increase of 1.5°C (ZAMG, 2020). In 2014, a nature reserve including 35 ha of meadows and pastures and 284 ha of forests (including open forests with interspersed abandoned grasslands) of the study area was established (Priller, 2019). To protect the species‐rich extensively used grasslands, restrictions on fertilizer application and mowing times apply within the nature reserve. Further, afforested former grasslands have been cleared on 14 ha, and put into traditional use again (Priller, 2019). Given that not all land owners have agreed to include their land into the reserve and that high‐conservation‐value habitats only make up a fraction of the study area, the reserve is split into several polygons (Figure 1).

### 2.2 Sampling design and data collection

A total of 145 relevés that were established in 1995 and 1996 by the last author of this study in grassland habitats in the Jaidhaus basin (Essl, 1998) were resurveyed in 2020 by the first author; relevés established in woody vegetation by Essl (1998) were excluded, as we focused the study on grassland vegetation. For relocating the relevés, field maps, old notations, and verbal descriptions of the plots in Essl (1998) were used, which provided accurate information on the localities of the relevés. Relocation was precise, with geographic uncertainty being 5–10 m. In the second survey, coordinates were collected by GPS. To reduce potential observer errors, the first and last author independently surveyed several plots jointly at the beginning of data collection in 2020.

The first survey was done using the Braun‐Blanquet method (Braun‐Blanquet, 1984), while the resurvey was done using species cover estimates in percent. For most relevés, plot size was 40 m^2^ (mostly squares of 5 m × 8 m depending on the terrain), and identical plot size was used in both surveys. Sampling took place in the main vegetation period between mid‐May and late August. The sampling date in the second survey was set close to the sampling date of the initial survey to ensure comparability. Species cover values were recorded separately for herb, shrub (height of woody plants more than 1 m), and tree layers (height more than 5 m). The scientific nomenclature of plant taxa is based on Fischer et al. (2008), while vegetation classes follow Mucina et al. (2016). Taxonomic harmonization across the survey data was done to ensure that taxa that have been identified at different taxonomic levels (e.g. species, subspecies) between survey periods are aligned; given that a taxon list of recorded species in the first survey was used in the second survey, only few data had to be harmonized taxonomically. To remove spatial biases by the few very large or small plots in our data set, we removed all plots smaller than 30 m^2^ and larger 50 m^2^, resulting in a reduced dataset of 138 plots.

### 2.3 Analysis

For analysis, we transformed the Braun‐Blanquet cover values to percentage values according to Tüxen and Ellenberg (1937) and van der Maarel (1979). Further, we retrieved the Ellenberg Indicator Values (EIVs) for all plant species from Ellenberg and Leuschner (2010). EIVs characterize species’ ecological preferences in relation to environmental factors, that is, nutrient, light and water availability, temperature, climatic continentality, soil reaction and soil salinity (Ellenberg & Leuschner, 2010). They are based on expert knowledge and are given in nine classes (except for water availability, where there are 12 classes). EIVs are widely used for ecological analyses (Ellenberg, 1992). Here, we used EIVs to calculate weighted community indices for each relevé (Käfe & Witte, 2004). For doing so, cover values of all species in a relevé were divided by the sum of all cover percentages of all occurring species with known EIV per relevé, and finally multiplied by the EIVs for the species. Summing up these values gives the community values of relevés for a certain environmental factor. Species with unknown EIV and species which behave indifferently regarding a certain EIV are not considered in this analysis. Community indices describe the ecological preferences of communities in relationship to certain environmental variables (Käfe & Witte, 2004; Gottfried et al., 2012); here, we analysed how community indices of paired relevés have changed over time. For comparison, we also ran the analyses with the unweighted community values. Results do not differ markedly, hence we provide the results for the analyses with weighted community values in the main manuscript and the results for the analyses with the unweighted community values in the supplement (Figure S2 and Table S6).

All relevés were assigned to phyotosociological vegetation classes according to Mucina et al. (2016). Further, for each relevé, information on land use (i.e. mown, grazed, abandoned) was recorded at both surveys. To test for changes in species number among vegetation classes and land‐use types between both survey periods, we did a test for normal distribution and afterwards a mean value comparison (Wilcoxon test, for matched samples). This was also done for the comparison of the weighted community values (nutrients, moisture, and temperature). Furthermore, we tested for differences in the community values between the two survey periods, grouped according to the vegetation classes. Therefore, we used a pairwise, non‐parametric test (Wilcoxon test). Land‐use changes among the two survey periods were visualized with a Sankey diagram. To investigate the effect of land‐use transitions between the two sampling periods on species richness, we ran linear models with a Gaussian error distribution. As response variable we used the change in species richness of a relevé between the sampling in 2020 and 1995 ($∆{\mathrm{SR}}_{2020-1995}$). As response variable we used the interaction term between the land‐use classification in 2020 and 1995. Model assumptions were checked visually using diagnostic plots. Finally, mean compositional similarity changes for each phytosociological vegetation class over time was calculated using the Jaccard dissimilarity index based on Baselga (2012). Additionally, we assessed the contribution of nestedness and turnover to compositional similarity (Baselga, 2012). Turnover represents the dissimilarity component due to species replacement, as compared to the dissimilarity component due to nestedness of species across time steps (Baselga, 2012). We used SPSS Statistic version 27 and the R software (R Core Team, 2019) for statistical analysis.

## 3 RESULTS

### 3.1 Changes in species richness

**FIGURE 2:**
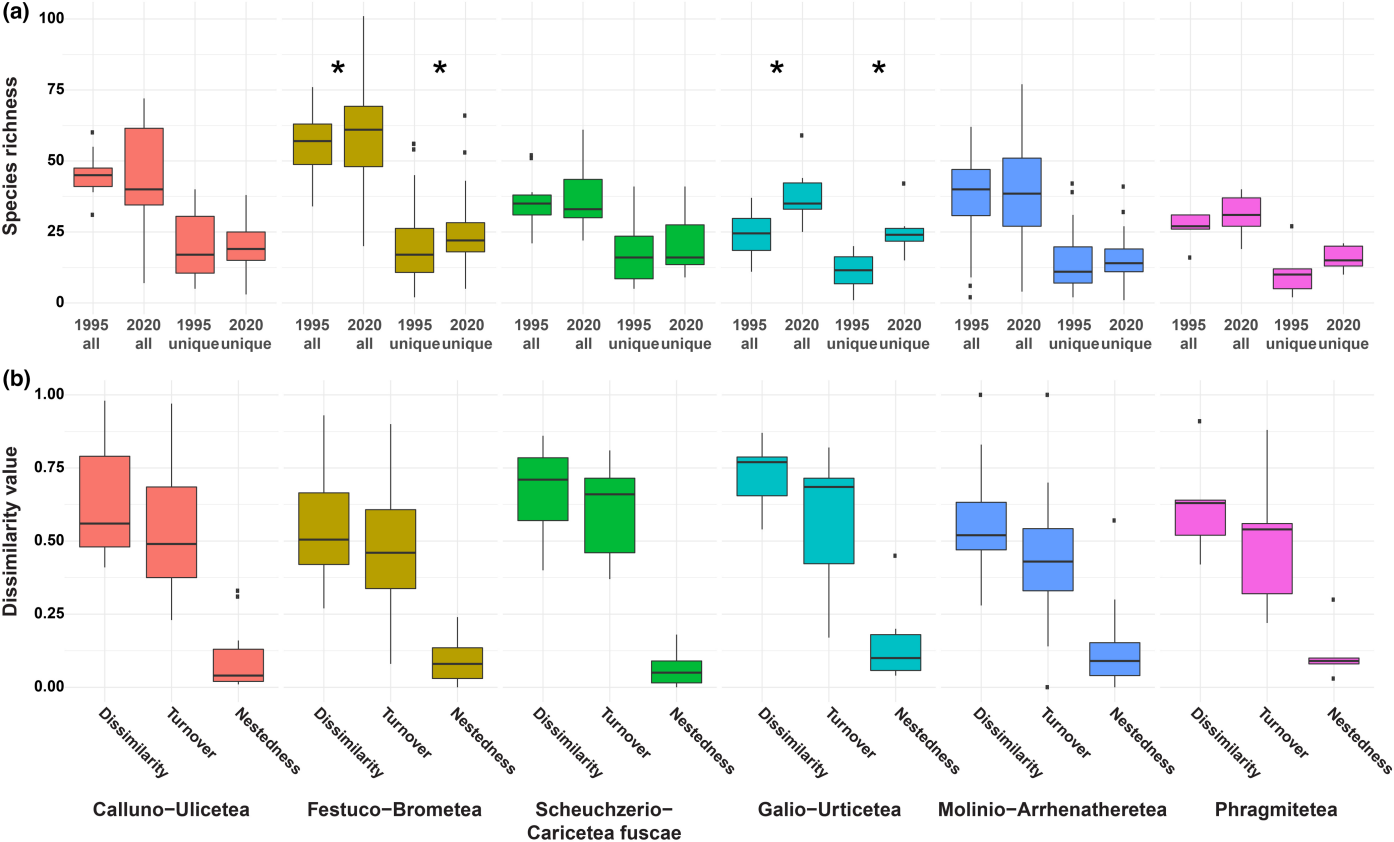
(a) Species richness changes across all plots within the different phytosociological vegetation classes between 1995 and 2020. Comparisons are given for overall species richness in 1995 (1995 all) and 2020 (2020 all) and for unique species in 1995 (1995 unique) to unique species in 2020 (2020 unique). (b) Dissimilarity of species composition between sites in 1995 and 2020 for all sites within the different phytosociological classes. Overall dissimilarity using the vJaccard index are provided as well as the separation into the different dissimilarity components (turnover and nestedness) following Baselga (2012).

A total of 421 species were recorded in 1995 in the 138 relevés, compared to 424 species in 2020. The median species richness per relevé across all relevés was 46 in the first survey period, and increased to 49 in the second one. In the most species‐rich relevé, 101 species were recorded in a plot with a size of 40 m^2^; in 2020, highest species richness was found in plots at sites that were recently managed for conservation purposes (i.e. restored nutrient‐poor grasslands that had formerly been afforested or abandoned) within the nature reserve. In these relevés, species of forests and forest fringes were abundant and contributed to high species richness. Median species richness across sites significantly increased from 1995 to 2020 for *Festuco‐Brometea* (55–61; Wilcoxon test: value = 665.5, *p*‐value < 0.05) and *Galio‐Urticetea* (24–32; Wilcoxon test: value = 0, *p*‐value < 0.05), but did not show any significant change for the other classes (Figure 2). When comparing the median number of unique species in sites of the different vegetation classes from 1995 and 2020, we again found significant increases in unique species richness for *Festuco‐Brometea* (17–22; Wilcoxon test: value = 665.5, *p*‐value < 0.05) and *Galio‐Urticetea* (12–24; Wilcoxon test: value = 0, *p*‐value < 0.05).

While mean species richness per relevé did moderately increase between both survey periods, we found pronounced changes in the frequency of occurrence of individual species (Table 1). The 10 species that lost the most occurrences between both survey periods represent species of nutrient‐poor grasslands such as *Carlina acaulis*. On the other hand, mainly species of nutrient‐rich grasslands (e.g. *Poa pratensis, Lolium perenne*) and ruderal sites belong to the winners.

**TABLE 1:** The top 10 losers and winners based on the change in number of occurrences in the relevés between the two sampling periods. Ellenberg Indicator Values are given for each species (T = temperature; M = moisture; N = nutrients)

Loser species	Decrease	T	M	N	Winner species	Increase	T	M	N
*Carlina acaulis*	−38	–	4	2	*Poa pratensis*	+34	–	5	6
*Centaurea jacea*	−31	–	–	–	*Fragaria vesca*	+24	–	5	6
*Euphrasia officinalis* ssp*. rostkoviana*	−31	–	–	4	*Lolium perenne*	+21	6	5	7
*Centaurea scabiosa*	−24	–	3	4	*Hypericum perforatum*	+21	6	4	4
*Campanula rotundifolia*	−23	5	–	2	*Senecio ovatus*	+20	–	5	8
*Gymnadenia conopsea*	−23	–	7	3	*Prunella vulgaris*	+20	5	5	5
*Briza media*	−22	–	–	2	*Mentha longifolia*	+20	5	8	7
*Potentilla erecta*	−22	–	–	2	*Festuca rupicola*	+20	7	3	2
*Buphthalmum salicifolium*	−21	–	4	3	*Arenaria serpyllifolia*	+20	–	4	–
*Tragopogon orientalis*	−20	–	5	6	*Galium aparine*	+19	6	–	8

While many moderately common species of nutrient‐poor grasslands declined, 55 species disappeared completely (Table S1). Unsurprisingly, most of the lost species were recorded only in a few plots in 1995, e.g. *Aster amellus*, *Crocus albiflorus* and *Eleocharis quinqueflora* (Essl, 1998). The loss of a species in the relevés does not necessarily imply that the respective species has become locally extinct in the study area. At the same time, 58 species were newly recorded in the second survey period. Apart from eight newly recorded alien species, several common species of forests like *Carex sylvatica*, *Galeobdolon montanum* or *Viola reichenbachiana* were recorded for the first time. Other newly recorded species show a preference for nutrient‐rich sites such as *Galium aparine* and *Clematis vitalba*.

### 3.2 Compositional similarity

Median dissimilarity of plots within phytosociological vegetation classes was ranging from 0.77 for *Galio‐Urticetea* to 0.51 for *Festuco‐Brometea*. Dissimilarity across classes was driven by species turnover (ranging from 0.69 to 0.54) compared to nestedness (ranging from 0.1 to 0.04) across all classes (Figure 2b; Table 2).

**TABLE 2:** Mean Jaccard dissimilarity measure for paired sites by time within the individual phytosociological vegetation classes. Additionally, the turnover and nestedness component of the dissimilarity measure is provided, following Baselga (2012).

Class	Dissimilarity	Turnover	Nestedness
*Calluno‐Ulicetea*	0.56	0.49	0.04
*Festuco‐Brometea*	0.51	0.46	0.08
*Scheuchzerio‐Caricetea fuscae*	0.71	0.66	0.05
*Galio‐Urticetea*	0.77	0.69	0.10
*Molinio‐Arrhenatheretea*	0.52	0.43	0.09
*Phragmitetea*	0.63	0.54	0.06

### 3.3 Changes in weighted community values in vegetation types over time

Overall, the numbers of relevés assigned to different vegetation classes changed between both survey periods (Table S2): the vegetation class with most relevés were semi‐dry grasslands of the *Festuco‐Brometea* (1995: 64 relevés, 2020: 54 relevés), followed by nutrient‐rich mesic grasslands of the *Molino‐Arrhenatheretea* (1995: 40 relevés, 2020: 41 relevés). Overall, the number of relevés declined for all vegetation classes of nutrient‐poor sites, while it increased for classes of nutrient‐rich sites.

**FIGURE 3:**
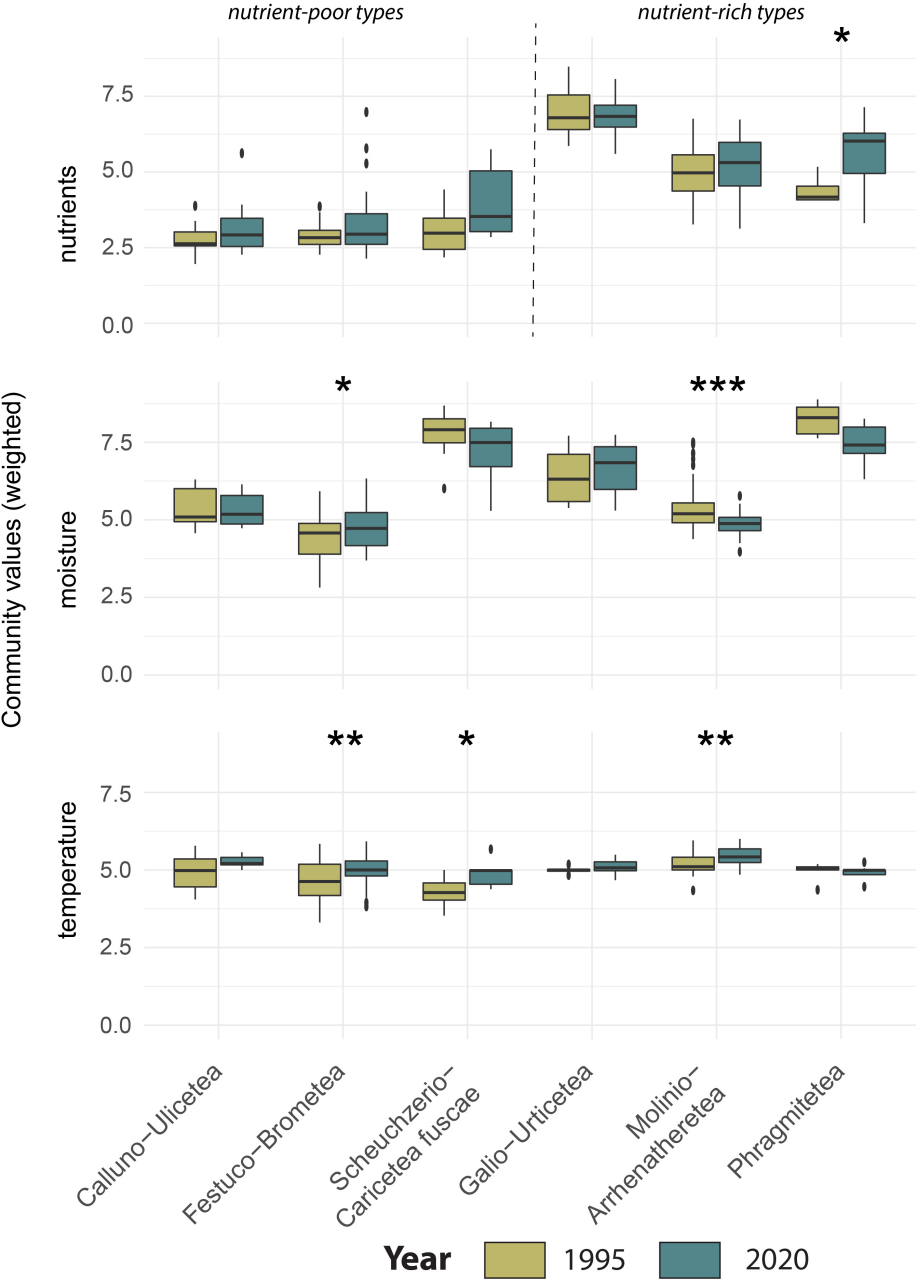
Weighted community values for nutrients (upper panel), moisture (middle panel) and temperature (lower panel) grouped into the most frequently recorded vegetation classes. Both the old and the new relevés belong to the vegetation classes corresponding to the data of 1995. *Calluno‐Ulicetea* (*N* = 11), *Festuco‐Brometea* (*N* = 64), *Galio‐Urticetea* (*N* = 6), *Molinio‐Arrhenatheretea* (*N* = 408), *Phragmitetea* (*N* = 5), *Scheuchzerio‐Caricetea fuscae* (*N* = 101). Outliers marked with a circle/star are located 1.5 times and 3 times respectively above the interquartile distance. Significant differences in marked classes: *, <0.05; **, <0.01 (Wilcoxon test, *p*‐value: 0.05).

Regarding the weighted community values for nutrients, a trend towards eutrophication over all vegetation classes is visible. The nutrient‐poor vegetation classes (i.e. *Festuco‐Brometea*, *Calluno‐Ulicetea*, *Scheuchzerio‐Caricetea fuscae*) show increasing trends of the weighted community values for nutrients in the mentioned classes between both surveys. For nutrient‐rich vegetation classes we observe a significant increase only for *Phragmitetea* (Figure 3 and Table S7). Similarly, there was a substantial change in weighted community values based on EIVs. For moisture, we observed a non‐significant trend towards more mesic conditions in vegetation classes of wet sites (i.e. *Phragmitetea* and *Scheuchzerio‐Caricetea fuscae*). However, we found a significant negative shift for *Molinio‐Arrhenatheretea* and a significant positive shift for *Festuco‐Brometea* (Figure 3 and Table S7). Regarding the weighted community values for temperature, there is a trend across all vegetation classes except *Phragmitetea* towards more thermophilic species composition over time. This trend is significant for *Festuco‐Brometea*, *Scheuchzerio‐Caricetea fuscae* and *Molinio‐Arrhenatheretea* (Figure 3 and Table S7).

### 3.4 Changes in land use and impact of establishing a nature reserve

Most resurveyed plots did not change in terms of their land‐use category between both surveys, but some changes occurred (Figure S3). Many of the fallows of the first survey (*n* = 15) were put into use again and most of these sites are now used as meadows or pastures. Considerably fewer plots (*n* = 3) were abandoned between both survey periods. Only a few sites, mostly already abandoned ones or mown grasslands in the first survey period, were afforested (two and two, respectively).

**FIGURE 4:**
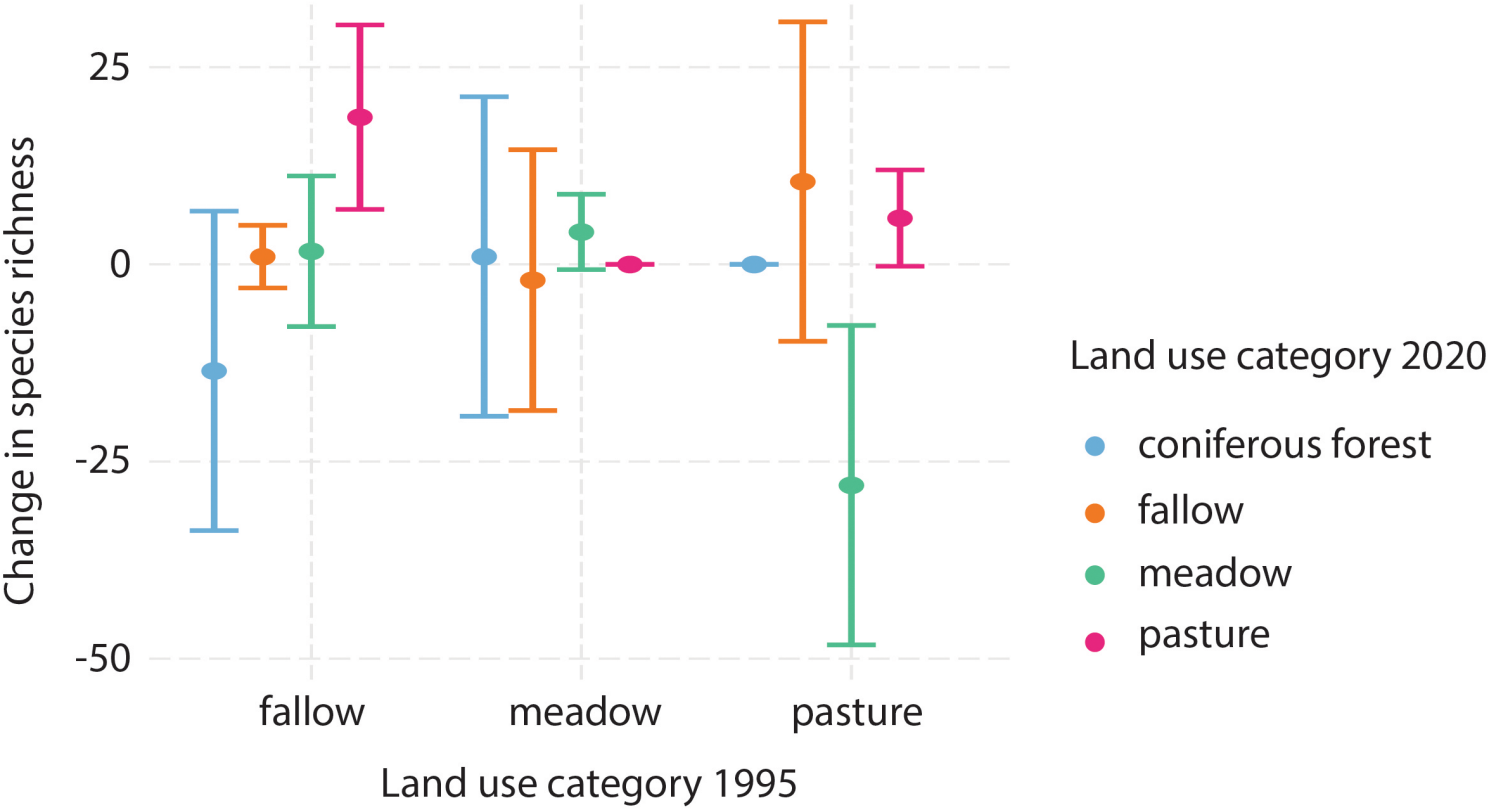
Changes in species richness due to land‐use transition effects between the first and second survey.

When analysing the effects of these land‐use transitions on species richness, we found a significant increase in species richness for land‐use transitions from fallow to pasture (estimate = 18.17, *p*‐value < 0.01) and a significant decrease in species richness for the transition from pasture to meadow (estimate = −28.00, *p*‐value < 0.01). Additionally, the models show a non‐significant increase in species richness in meadows (estimate = 4.08, *p*‐value = 0.09) and pastures (estimate = 5.73, *p*‐value = 0.06). Overall, the model explains only 11% of the variation in the data, indicating that other factors not sampled in this study explain a large fraction of changes in species richness. Additionally, for several transitions the number of replicates is too small to derive significant results: e.g., pasture to meadow (*n* = 2; Figure 4).

Within the protected area, grasslands are mainly used as meadows and pastures, and a considerable part is abandoned. However, the majority of the fallows is located outside the protected area, as are the ruderal sites (Figure S4). In addition, the plots of the first and second survey period within the protected area differ significantly in mean plant‐species richness. In particular, weighted community values for nutrients have significantly increased for relevés of several vegetation classes outside the protected area (*Festuco‐Brometea*, *Calluno‐Ulicetea*, *Scheuchzerio‐Caricetea fuscae*), whereas no such change occurred within the protected area. In terms of land use, fallow land and pastures outside the protected area belong to the categories most affected by increase in EIV for nutrients.

### 3.5 Alien species

The number of alien species has increased from three species recorded in 1995 to 11 species recorded in 2020; in addition, the number of occurrences of alien species has increased. In the first sampling period, five occurrences of alien species were recorded, while this value increased to 48 in 2020. The most common alien species in the study area in 2020 was *Erigeron annuus*, which was recorded in 14 plots (Table S3). Alien species were mostly recorded in plots of the *Molinio‐Arrhenateretea*, *Stellarietea* and *Galio‐Urticetea* (Tables S3 and S4). Land‐use categories such as ruderal sites and fallows are more often invaded by alien species than sites with a regular disturbance regime (mowed and grazed areas).

## 4 DISCUSSION

### 4.1 Changes in species richness and species composition over time

With a total number of 421 species in the initial survey in 1995 and 424 species recorded in 2020, there is a slight increase in the total recorded species number. Similarly, mean species numbers in plots only changed little and changes were not significant in all vegetation classes. These findings are in contrast to other resurvey studies that revealed mean plant‐species richness declines in grasslands of many regions of Central Europe (e.g., Wesche et al., 2012; Hülber et al., 2017; Jansen et al., 2019; Bruelheide et al., 2020; Eichenberg et al., 2021). A study from Denmark that analysed vegetation changes over more than 100 years in a broad range of habitat types also showed an increase in mean species richness, however, that was mostly attributed to increases in alien species richness outweighing a simultaneous decline in native species richness (Finderup Nielsen et al., 2019).

In the present study, species‐richness changes were marginal in most plots, with substantial increases or decreases in some others. At the same time, we observed substantial changes in species composition between both sampling periods leading to a strong signal of species turnover instead of nestedness as driver of compositional dissimilarity. Regarding the newly recorded species in resurveyed relevés, these are mainly species which prefer shadier sites. This finding can be attributed to the ongoing succession of abandoned sites, where encroachment of shrubs and trees is responsible for reduction in light availability. The newly recorded species also include several alien species, which is in accordance with other studies (Finderup Nielsen et al., 2019; Eichenberg et al., 2021). While alien species richness and abundance increased, some native species disappeared completely. Species that have disappeared since the first survey are mostly species of nutrient‐poor sites and species which were already rare in 1995 (Essl, 1998). Changes in land use such as intensification and fertilization, loss and change of habitats are the main causes for the decline of rare and threatened species in Central European grasslands (e.g., Kempel et al., 2020; Zehm et al., 2020). Further, the conspicuous decline of moderately common plants of nutrient‐poor grasslands (Table 1) shows that widespread eutrophication has affected many plots. Several of these species are already listed as near threatened in the regional Red List (Hohla et al., 2009). Species suffering the highest losses prefer nutrient‐poor sites, which is in line with other studies from Central Europe. Jansen et al. (2019) and Bruelheide et al. (2020), for example, show that moderate common species of dry to wet habitats decreased the most in recent decades in Germany. In grasslands of the biosphere reserve Wienerwald, a similar set of species of nutrient‐poor sites declined conspicuously (Hülber et al., 2017). Recently, Staude et al. (2022) reported changes in the vegetation of European habitats (trajectories across lowland grasslands, forests and mountain summits) towards a more nutrient‐demanding species composition.

Conversely, the species that have increased most in occurrences are predominantly species of nutrient‐rich sites (Table 1). These findings are again in line with the results of another resurvey study from Austria (Hülber et al., 2017) and a study from Europe (Staude et al., 2022). Further, a study from Germany shows an increase of indifferent species and species of agricultural grasslands in nutrient‐poor acidic *Nardus* grasslands (Peppler‐Lisbach et al., 2020).

### 4.2 Changes of weighted community values over time

A eutrophication trend is evident across all vegetation classes as shown by the rise in weighted community indices for nutrients — particularly for plots of nutrient‐poor vegetation classes and for plots located outside the protected areas. Hülber et al. (2017) observed the same trend for oligotrophic habitats, like *Brometalia*. In this study, we also found a significant trend of increasing nutrient community values in the class of *Festuco‐Brometea*.

In the absence of data for relevant drivers of nutrient supply we can only speculate about the reasons of this change. A likely driver is atmospheric nitrogen deposition that is known as an important cause for the eutrophication of nutrient‐poor habitats (e.g. Bobbink et al., 2010; Pannek et al., 2015). However, for calcareous grasslands in northwestern Germany, no effect of atmospheric nitrogen deposition on species richness was found (Diekmann et al., 2014). Another line of argument relates to the intensification of land use and associated increases in fertilizer application that lead to higher nutrient availability. Numerous studies from Central Europe show evidence for the negative impact of land‐use intensification on oligotrophic species and their habitats (e.g. Diekmann et al., 2014; Halada et al., 2017; Jansen et al., 2019; Peppler‐Lisbach et al., 2020; Zehm et al., 2020). Finally, in abandoned grasslands endogenous eutrophication may occur as in the absence of human land use no nutrients are removed in the hay or by grazing.

The findings of Gossner et al. (2016) show an increase of generalistic species typical for intensively used grasslands in Germany. While it is well known that traditional land use is important for the maintenance of semi‐natural grasslands, intensification leads to the opposite (Diekmann et al., 2014; Halada et al., 2017). The reason why changes in land use have a minor importance in explaining changes in species richness in the present study could be due to the coarse classification of the land‐use categories. Conceivably, a differentiation between extensive and intensive mowing or grazing, would have given better insights.

Land‐use intensification is not only associated with changes in weighted community values for nutrients, but may also have knock‐on effects on the water supply of wet grasslands. Several of the wet grasslands were partly drained between both survey periods, and accordingly, weighted community values for moisture of the plots of *Phragmitetea* became significantly less wet. Many of the rare wet habitats in the study area were already drained before the first investigation (Essl, 1998). Similarly to our results, the decline and degradation of wet habitats during recent decades can be observed in many regions of Central Europe (Kempel et al., 2020).

The significant increases in the community indices for temperature across vegetation classes provides evidence for thermophilization by climate change. This finding is widely supported by other studies of lowland (e.g. Martin et al., 2019) to alpine habitats (e.g. Gottfried et al., 2012) in Central Europe. Warm‐adapted species increased relatively while cold‐adapted ones declined during the study period.

### 4.3 Effects caused by establishing a protected area

In 2014, a protected area was established in a part of the study area, and a total of 48 plots are located within it. Since then, substantial conservation actions have been carried out there including the removal of 14 ha of forests and overgrown abandoned grasslands, and the reestablishment of extensive land use (mowing, grazing) in these areas (Priller, 2019). Accordingly, 19 plots — 12 of which are located in the protected area — that had been abandoned in 1995 were in use again in 2020.

Although the protected area was established nearly 20 years after the first survey — and thus conservation actions have only been established recently — differences in the trajectories of species change with the relevés located outside the protected area can already be identified. In particular, community values for nutrients within the protected area did not increase significantly, while this was the case in the plots outside the protected area. Thus, it can be inferred that the management of the protected area has positive effects in halting eutrophication.

Several studies have shown that well‐managed protected areas are able to halt — or reverse — species declines that are occurring in not‐protected landscapes. For instance, a Bavarian study (Zehm et al., 2020) shows that the decline of rare species outside protected areas is much stronger than inside protected areas. Still, protected areas are not large enough and not managed well enough to halt species declines altogether (Kempel et al., 2020). As a consequence, further measures have to be taken.

### 4.4 The spread of alien species (and effects of climate change)

In addition to major changes in the species composition of the native species, the number of alien species and their abundance has also changed during the 25 years elapsed between both surveys. The number of alien species has increased from three (with a total of five occurrences) to 11 species (with a total of 48 occurrences) species. Still, the level of invasions is low with 2.6% of all species being alien, and 0.7% of all occurrences in the plots being alien species. This is also evident when compared to many regions in Europe, where alien species currently play a much bigger role in vegetation change analyses by resurveyed relevés and (partly) outweigh the decline of native species (e.g., Finderup Nielsen et al., 2019; Eichenberg et al., 2021). Likely, one reason for the low level of invasion is that the study area is relatively distant to areas with high anthropogenic pressures (e.g., large settlements, large roads) that are associated with high propagule pressure and, consequently, higher abundances of invaders (Medvecká et al., 2013). Furthermore, the climatic conditions of the rather cool pre‐alpine valley could have reduced the increase of alien species, as most alien species in Central Europe are confined to warm lowlands (Chytrý et al., 2009). With increasing altitude, the number of alien species is known to decrease drastically (Kueffer, 2011; Vorstenbosch et al., 2020). Nevertheless, more alien species are projected to migrate to higher altitudes in the future and expand into the montane and subalpine regions under further climate change (Becker et al., 2005; Alexander et al., 2011, Kueffer,  2011). Further, alien species in Central Europe prefer mostly nutrient‐rich habitats (Kowarik, 2010; Jansen et al., 2011; Medvecká et al., 2013). Accordingly, aliens occur mostly in plots of nutrient‐rich grasslands such as *Molinio‐Arrhenatheretea*, *Stellarietea* and *Galio‐Urticetea*.

The most common alien species in the study area, *Erigeron annuus* (14 plots), is a widespread species in Austria (Fischer et al., 2008). For example, vegetation surveys in semi‐dry grasslands in the lowlands of Upper Austria recorded this species as the most frequent alien (Essl & Dirnböck, 2008). The closely related *Conyza canadensis*, another widespread alien species, prefers ruderal sites also (Kowarik, 2010). Even though the richness and abundance of invaders is still low, it can be assumed that the level of invasion in the study area will increase in the future. Finally, it is well known that climate change will facilitate the spread of alien species (Kowarik, 2010). With increasing temperatures even more regions will become suitable for alien species (Kleinbauer et al., 2010).

## 5 CONCLUSIONS

While we found that local species richness as documented in relevés increased moderately during 25 years, we found substantial changes in species composition and pronounced species turnover; in particular, we found substantial changes in community indices, particularly for nutrients, temperature, and water. In addition, alien species have spread considerably between both survey periods. Finally, we found different trajectories in vegetation changes between plots located inside and outside of a recently established protected area, providing evidence of the effectiveness of conservation measures associated with this conservation area. For instance, there were substantial differences in the trajectories of community values for nutrients of plots inside the protected areas compared to those located outside. Overall, this study shows that species‐rich grasslands are under substantial pressure in Central Europe, even in locations like the Jaidhaus basin, where substantial conservation efforts have been carried through recently. The different trajectories of vegetation change inside and outside the nature reserve underline that dedicated conservation measures (such as restoration of abandoned grasslands) are essential for the maintenance of nutrient‐poor, species‐rich grasslands.

## AUTHOR CONTRIBUTIONS

Franz Essl and Helena Schwaiger conceived the study, Helena Schwaiger did the fieldwork, and led the analyses. Bernd Lenzer contributed to the analyses. Helena Schwaiger led writing, Franz Essl and Bernd Lenzer contributed to it.

## Supporting information


**Figure S1** Aerial photographs of the study area and the nature reserve (red line) taken in 1953 (A) (source: Bundesamt für Eich‐ und Vermessungswesen, flight title: 1953002, picture 8060 edited by Christian Hatzenbichler) and in 2013 (B) (source: http://www.doris.at/Karten/karten.aspx edited by Christian Hatzenbichler).
**Figure S2** Unweighted community values for nutrients (upper panel), moisture (middle panel) and temperature (lower panel) grouped into the most frequently recorded vegetation classes.
**Figure S3** Land‐use changes over 25 years.
**Figure S4** Land use of the relevés within and outside the protected area.
**Table S1** Overview of vascular plant species that have been recorded in the first survey only and of vascular plant species that have been recorded in the second survey only.
**Table S2** Number of relevés per phytosociological class for the two sampling periods.
**Table S3** Number of occurrences of alien species in the 138 relevés in both sampling periods.
**Table S4** Number of neophytes per phytosociological class in both sampling periods.
**Table S5** Number of neophytes per land‐use type in both sampling periods.
**Table S6** Results from the pairwise Wilcoxon test of the unweighted community values for temperature, moisture and nutrients between the two sampling periods.
**Table S7** Results from the pairwise Wilcoxon test of the weighted community values for temperature, moisture and nutrients between the two sampling periods.

## Data Availability

The data that support the findings of this study have been submitted to ReSurveyEurope (http://euroveg.org/eva‐database‐resurvey‐europe) and are available according to the ReSurveyEurope rules of access.
