# Supplementary material for: No species loss, but pronounced species turnover in grasslands in the Northern Alps over 25 years
Source: Appl Veg Sci. 2022 Dec 18;25(4):e12700. doi: 10.1111/avsc.12700 (PMC10107162; doi:10.1111/avsc.12700)
Supplement: Supplementary file 1 — Figure S1 Aerial photographs of the study area and the nature reserve (red line) taken in 1953 (A) (source: Bundesamt für Eich‐ und Vermessungswesen, flight title: 1953002, picture 8060 edited by Christian Hatzenbichler) and in 2013 (B) (source: http://www.doris.at/Karten/karten.aspx edited by Christian Hatzenbichler). Figure S2 Unweighted community values for nutrients (upper panel), moisture (middle panel) and temperature (lower panel) grouped into the most frequently recorded vegetation classes. Figure S3 Land‐use changes over 25 years. Figure S4 Land use of the relevés within and outside the protected area. Table S1 Overview of vascular plant species that have been recorded in the first survey only and of vascular plant species that have been recorded in the second survey only. Table S2 Number of relevés per phytosociological class for the two sampling periods. Table S3 Number of occurrences of alien species in the 138 relevés in both sampling periods. Table S4 Number of neophytes per phytosociological class in both sampling periods. Table S5 Number of neophytes per land‐use type in both sampling periods. Table S6 Results from the pairwise Wilcoxon test of the unweighted community values for temperature, moisture and nutrients between the two sampling periods. Table S7 Results from the pairwise Wilcoxon test of the weighted community values for temperature, moisture and nutrients between the two sampling periods. [file AVSC-25-0-s001.docx]

**No species loss, but pronounced species turnover in grasslands in the Northern Alps over 25 years**

Helena Schwaiger, Bernd Lenzer^1,#^, Franz Essl^1,*, #,^

^1^ BioInvasions. Global Change. Macroecology Group, Department of Botany and Biodiversity Research, University of Vienna, Austria

*Corresponding author: [franz.essl@univie.ac.at](mailto:franz.essl@univie.ac.at)

^#^These two authors serve as joint senior authors.

**Supplementary Online Material**


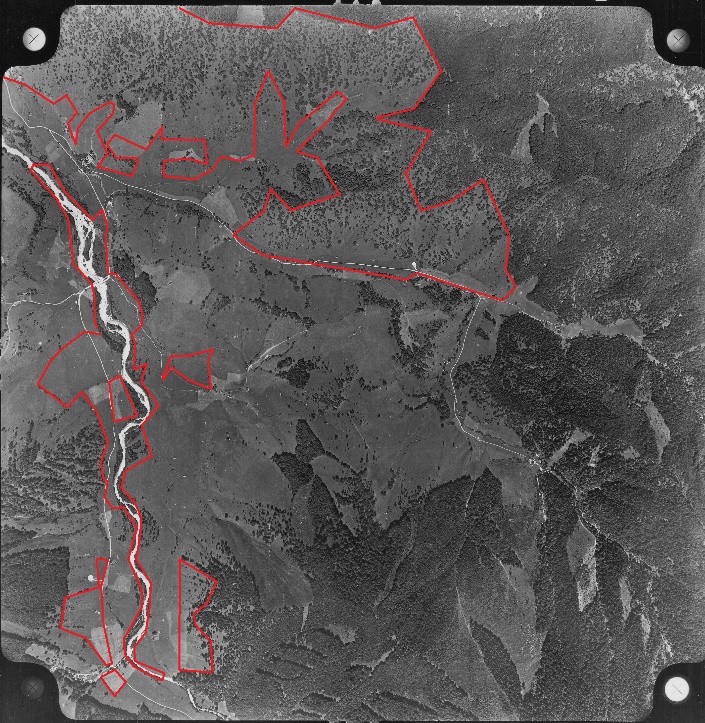

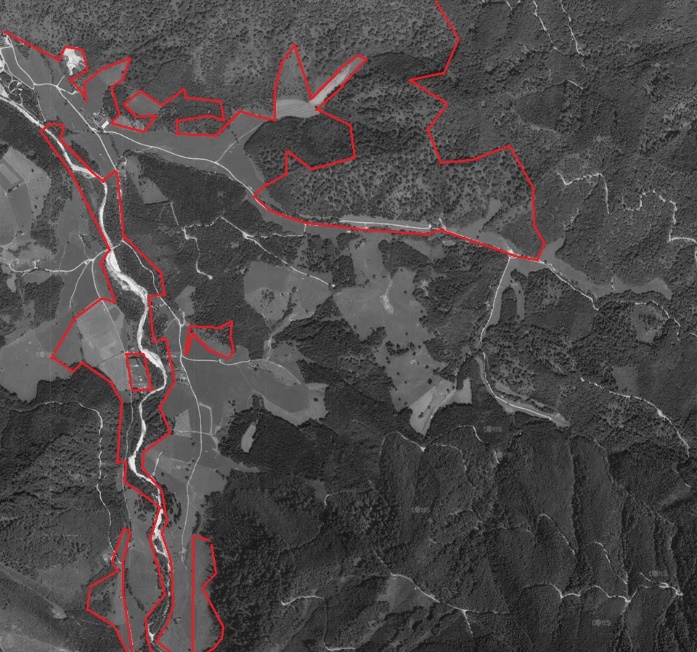


**A**

**B**

Figure S1: Aerial photographs of the study area and the nature reserve (red line) taken in 1953 (A) (source: Bundesamt für Eich- und Vermessungswesen, flight title: 1953002, picture 8060 edited by Christian Hatzenbichler) and in 2013 (B) (source: http://www.doris.at/Karten/karten.aspx edited by Christian Hatzenbichler).


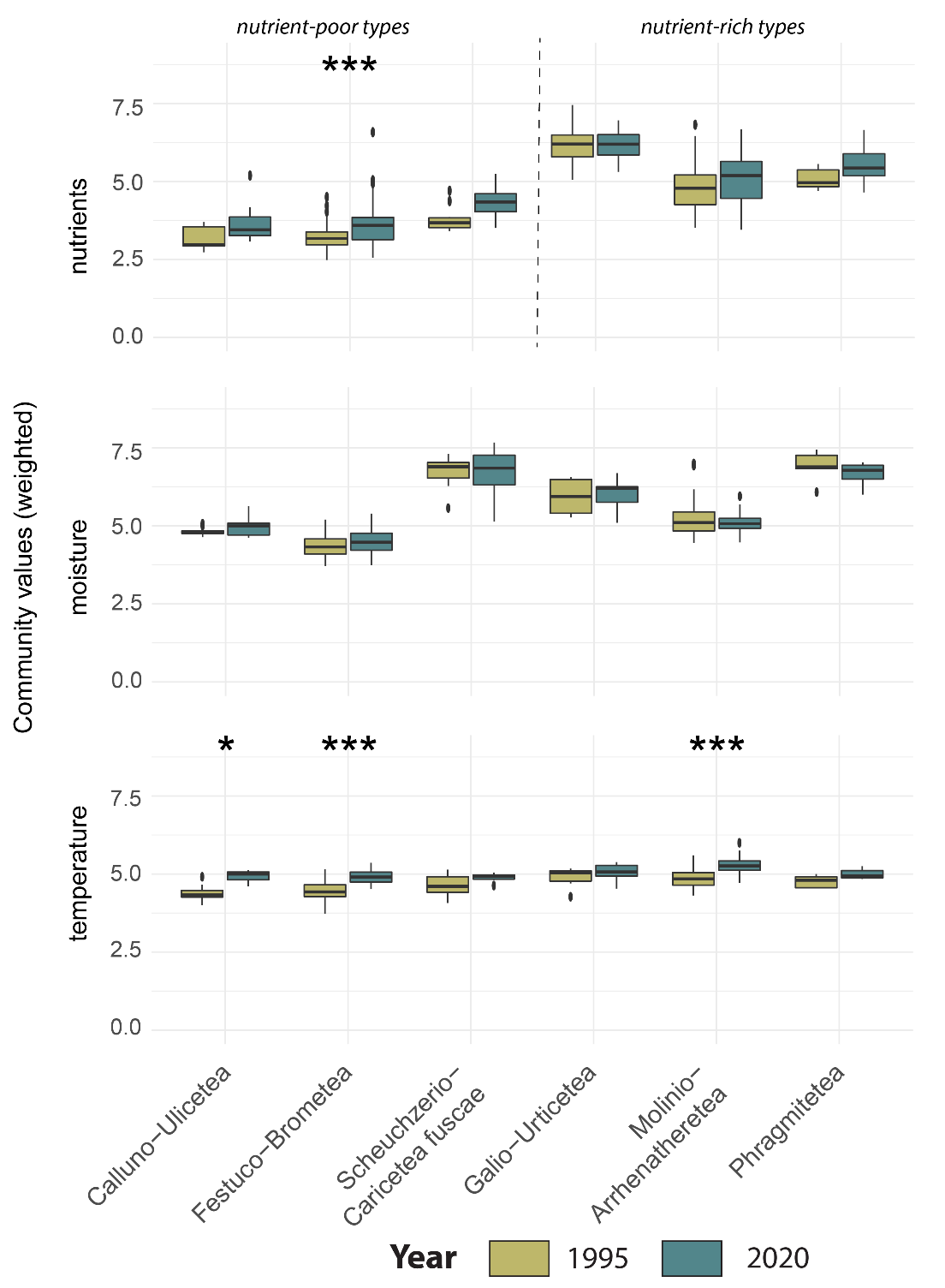


Figure S2: Unweighted community values for nutrients (upper panel), moisture (middle panel) and temperature (lower panel) grouped into the most frequently recorded vegetation classes. Both the old and the new relevés belong to the vegetation classes corresponding to the data of 1995. Calluno-Ulicetea (N=11), Festuco-Brometea (N=64), Galio-Urticetea (N=6), Molinio-Arrhenatheretea (N=40), Phragmitetea (N=5), Scheuchzerio-Caricetea fuscae (N=10). Outliers marked with a circle are located 1.5 times respectively 3 times above the interquartile distance. Significant differences in marked classes: * < 0.05; ** < 0.01 (Wilcoxon-Test, p-value: 0.05)


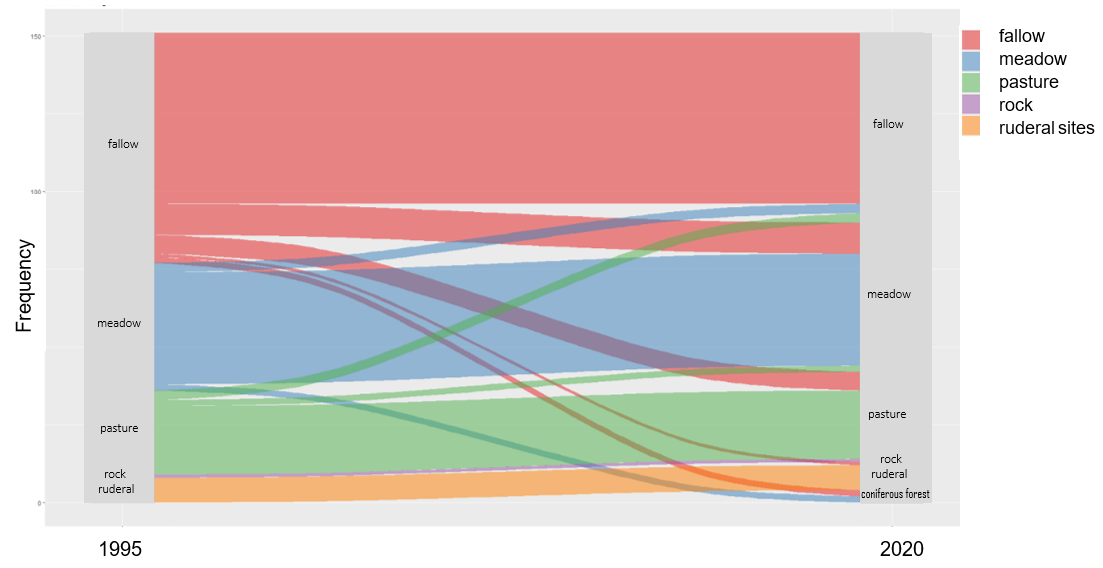


Figure S3: Land use changes over 25 years. Shown are the transitions between different land use types.


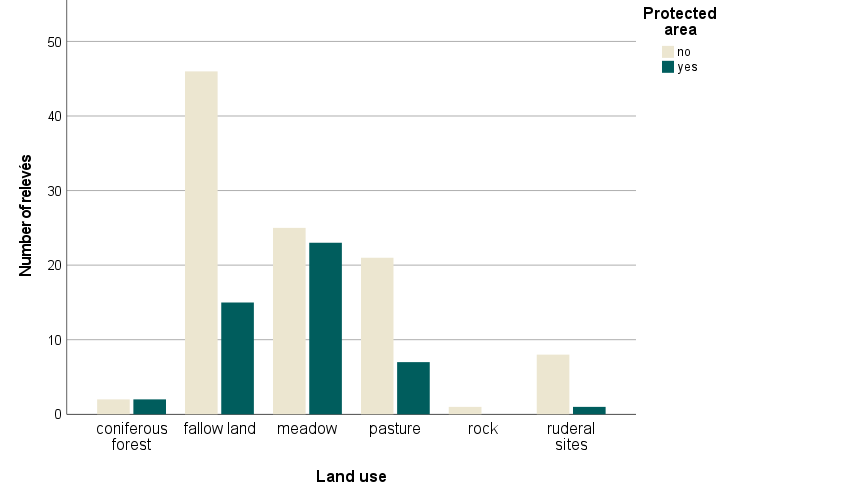


Figure S4: Land use of the relevés within and outside the protected area. Only the land use of the second survey period (2020) is shown.

Table S1: Overview on vascular plant species that have been recorded in the first survey only and on vascular plant species that have been recorded in the second survey only. Alien species are highlighted (orange).

| **Species recorded only in 1^st^ survey** | **Number of relevés** | **Species recorded only in 2^st^ survey** | **Number of relevés** |
| --- | --- | --- | --- |
| *Antennaria dioica* | 5 | *Acer campestre* | 1 |
| *Asplenium trichomanes* | 1 | *Actaea spicata* | 2 |
| *Asplenium viride* | 1 | *Agrimonia eupatoria* | 1 |
| *Aster amellus* | 3 | *Alopecurus pratensis* | 10 |
| *Athamanta cretensis* | 1 | *Aruncus dioicus* | 1 |
| *Betula pendula* | 2 | *Athyrium filix-femina* | 5 |
| *Bidens tripartita* | 1 | *Calystegia sepium* | 1 |
| *Calycocorsus stipitatus* | 1 | *Cardamine trifolia* | 1 |
| *Campanula cespitosa* | 1 | *Carex muricata* agg. | 6 |
| *Campanula glomerata* | 1 | *Carex sylvatica* | 17 |
| *Carex sempervirens* | 2 | *Clematis vitalba* | 12 |
| *Carex spicata* | 3 | *Crataegus laevigata* | 1 |
| *Cerastium carinthiacum* ssp. *carinthiacum* | 1 | *Dactylorhiza maculata* s.lat. (inkl. *fuchsii*) | 5 |
| *Crepis alpestris* | 12 | *Dentaria bulbifera* | 2 |
| *Crepis praemorsa* | 2 | *Digitalis grandiflora* | 1 |
| *Crocus albiflorus* | 2 | *Dryopteris filix-mas* | 4 |
| *Dactylorhiza majalis* | 3 | *Elymus caninus* | 1 |
| *Dryopteris carthusiana* | 1 | *Epilobium ciliatum* | 4 |
| *Eleocharis quinqueflora* | 1 | *Epilobium montanum* | 2 |
| *Equisetum fluviatile* | 1 | *Erigeron annuus* | 14 |
| *Euphrasia stricta* | 1 | *Euphorbia amygdaloides* | 2 |
| *Fallopia convolvulus* | 1 | *Festuca arundinacea* | 1 |
| *Gentianella aspera* | 15 | *Festuca gigantea* | 1 |
| *Geranium sanguineum* | 2 | *Galeobdolon montanum* | 1 |
| *Glyceria notata* | 1 | *Galium aparine* | 19 |
| *Heracleum austriacum* | 2 | *Gymnocarpium robertianum* | 3 |
| *Hieracium bauhinii* | 1 | *Hedera helix* | 1 |
| *Hieracium piloselloides* | 1 | *Hepatica nobilis* | 6 |
| *Hieracium sabaudum* | 1 | *Impatiens glandulifera* | 4 |
| *Hypericum montanum* | 3 | *Impatiens parviflora* | 5 |
| *Hypochoeris maculata* | 12 | *Lamium purpureum* | 1 |
| *Lembotropis nigricans* | 3 | *Lunaria rediviva* | 2 |
| *Lilium bulbiferum* | 1 | *Luzula multiflora* s.lat. | 1 |
| *Lycopodium clavatum* | 1 | *Lysimachia nummularia* | 8 |
| *Lysimachia nemorum* | 1 | *Matricaria chamomilla* | 1 |
| *Mentha arvensis* | 1 | *Moehringia muscosa* | 2 |
| *Ononis spinosa* | 2 | *Orobanche flava* | 3 |
| *Ophioglossum vulgatum* | 1 | *Panicum capillare* | 3 |
| *Orchis mascula* | 3 | *Paris quadrifolia* | 2 |
| *Orobanche reticulata* | 1 | *Phyteuma spicatum* | 2 |
| *Persicaria hydropiper* | 1 | *Polygala amara* | 13 |
| *Persicaria maculosa* | 1 | *Polygonatum multiflorum* | 3 |
| *Persicaria vivipara* | 1 | *Polygonatum verticillatum* | 1 |
| *Pinus sylvestris* | 1 | *Polypodium vulgare* | 1 |
| *Potentilla heptaphylla* | 1 | *Prenanthes purpurea* | 1 |
| *Primula vulgaris* | 1 | *Pulmonaria officinalis* | 3 |
| *Prunus avium* | 1 | *Rubus fruticosus* agg. | 6 |
| *Pyrola rotundifolia* | 2 | *Sanicula europaea* | 2 |
| *Ranunculus aconitifolius* | 1 | *Solidago canadensis* | 1 |
| *Rosa pendulina* | 2 | *Solidago gigantea* | 2 |
| *Salix caprea* | 4 | *Sonchus asper* | 10 |
| *Selinum carvifolia* | 1 | *Sonchus oleraceus* | 1 |
| *Sherardia arvensis* | 1 | *Ulmus glabra* | 1 |
| *Tephroseris crispa* | 2 | *Verbascum nigrum* | 1 |
| *Thymus praecox* ssp. *praecox* | 1 | *Veronica hederifolia* agg. | 1 |
|  |  | *Veronica persica* | 1 |
|  |  | *Viola mirabilis* | 2 |
|  |  | *Viola reichenbachiana* | 3 |

Table S2: Number of relevés per phytosociological class for the two sampling periods

| **Class** | **# relevés**  **1995** | **# relevés**  **2020** |
| --- | --- | --- |
| Calluno-Ulicetea | 11 | 7 |
| Festuco-Brometea | 64 | 54 |
| Scheuchzerio-Caricetea fuscae | 10 | 7 |
| Galio-Urticetea | 6 | 10 |
| Molinio-Arrhenatheretea | 40 | 41 |
| Phragmitetea | 5 | 9 |

Table S3: Number of occurrences of alien species in the 138 relevés at both sampling periods

| **Species** | **# relevés**  **1995** | **# relevés**  **2020** |
| --- | --- | --- |
| *Conyza canadensis* | 1 | 6 |
| *Epilobium ciliatum* | 0 | 4 |
| *Erigeron annuus* | 0 | 14 |
| *Galinsoga ciliata* | 2 | 4 |
| *Impatiens glandulifera* | 0 | 4 |
| *Impatiens parviflora* | 0 | 5 |
| *Juncus tenuis* | 2 | 4 |
| *Panicum capillare* | 0 | 3 |
| *Solidago canadensis* | 0 | 1 |
| *Solidago gigantea* | 0 | 2 |
| *Veronica persica* | 0 | 1 |

Table S4: Number of neophytes per phytosociological class at both sampling periods

| **Class** | **# neophytes**  **1995** | **# neophytes**  **2020** |
| --- | --- | --- |
| Calluno-Ulicetea | 0 | 4 |
| Festuco-Brometea | 0 | 2 |
| Scheuchzerio-Caricetea fuscae | 0 | 2 |
| Galio-Urticetea | 0 | 8 |
| Molinio-Arrhenatheretea | 4 | 21 |
| Phragmitetea | 0 | 2 |
| Stellarietea | 1 | 9 |

Table S5: Number of neophytes per land use type at both sampling periods

| **Class** | **# neophytes**  **1995** | **# neophytes**  **2020** |
| --- | --- | --- |
| Ruderal | 4 | 17 |
| Pasture | 1 | 7 |
| Meadow | 0 | 6 |
| Coniferous forest | 0 | 2 |
| Fallow | 0 | 16 |

Table S6: Results from the pairwise Wilcoxon-Test of the unweighted community values for temperature, moisture and nutrients between the two sampling periods. Results are given for the most frequently recorded vegetation classes corresponding to the data of 1995. Chi sq = Chi squared, df = degrees of freedom.

|  | **Temperature** | | | **Moisture** | | | **Nutrients** | | |
| --- | --- | --- | --- | --- | --- | --- | --- | --- | --- |
|  | Chi sq | df | p-value | Chi sq | df | p-value | Chi sq | df | p-value |
| Calluna-Ulicetea | 9.76 | 1 | 0.002 | 0.35 | 1 | 0.555 | 3.12 | 1 | 0.077 |
| Festuco-Brometea | 58.06 | 1 | 0 | 4.01 | 1 | 0.45 | 11.62 | 1 | 0.001 |
| Molinio-Arrhenatheretea | 31.5 | 1 | 0 | 0.16 | 1 | 0.691 | 2.58 | 1 | 0.108 |
| Scheuchzerio-Caricetea fuscae | 2.44 | 1 | 0.118 | 0.01 | 1 | 0.922 | 3.63 | 1 | 0.057 |
| Galio-Urticetea | 0.96 | 1 | 0.328 | 0.11 | 1 | 0.745 | 0.05 | 1 | 0.828 |
| Pharagmitetea | 3.49 | 1 | 0.062 | 1.14 | 1 | 0.285 | 1.28 | 1 | 0.257 |

Table S7: Results from the pairwise Wilcoxon-Test of the weighted community values for temperature, moisture and nutrients between the two sampling periods. Results are given for the most frequently recorded vegetation classes corresponding to the data of 1995. Chi sq = Chi squared, df = degrees of freedom.

|  | **Temperature** | | | **Moisture** | | | **Nutrients** | | |
| --- | --- | --- | --- | --- | --- | --- | --- | --- | --- |
|  | Chi sq | df | p-value | Chi sq | df | p-value | Chi sq | df | p-value |
| Calluna-Ulicetea | 1.49 | 1 | 0.221 | 0.05 | 1 | 0.821 | 0.9 | 1 | 0.342 |
| Festuco-Brometea | 8.1 | 1 | 0.004 | 3.85 | 1 | 0.05 | 1.7 | 1 | 0.192 |
| Molinio-Arrhenatheretea | 8.01 | 1 | 0.005 | 10.93 | 1 | 0.001 | 1.47 | 1 | 0.225 |
| Scheuchzerio-Caricetea fuscae | 4.83 | 1 | 0.028 | 1.37 | 1 | 0.242 | 2.44 | 1 | 0.118 |
| Galio-Urticetea | 1.18 | 1 | 0.277 | 0.42 | 1 | 0.515 | 0.05 | 1 | 0.828 |
| Pharagmitetea | 1.3 | 1 | 0.255 | 4.48 | 1 | 0.028 | 3.74 | 1 | 0.053 |
